# Resilience and Quality of Life: The Role of Positive Affect on Healthcare Professionals in High‐Stress Environments

**DOI:** 10.1111/wvn.70176

**Published:** 2026-09-21

**Authors:** Nurit Zusman, Martine Denise Freeman, Caryn Scheinberg Andrews

**Affiliations:** ^1^ Henrietta Szold Hadassah School of Nursing at the Faculty of Medicine at Hebrew University Ein Kerem Jerusalem Israel

## Abstract

**Background:**

Healthcare professionals (HCPs) operate within environments of chronic occupational stress; a burden significantly amplified in Israel by the compounded pressures of the COVID‐19 pandemic and active warfare. While resilience and positive affect (PA) are established protective factors, the nature of their interactive role in sustaining quality of life (QoL) during prolonged, extreme crises remains under‐explored.

**Aim:**

This study examines resilience, positive affect (PA), and quality of life (QoL) among healthcare professionals (HCPs), investigating whether PA moderates the relationship between resilience and QoL during a prolonged crisis.

**Methods:**

A cross‐sectional national online survey was conducted among 260 Israeli HCPs nationally. Participants completed validated instruments including the Brief Resilience Scale (BRS), the Positive and Negative Affect Schedule (PANAS), and the Multicultural Quality of Life Index (MQLI). Moderation analyses assessed the predictive effects of resilience and the moderating role of PA on QoL.

**Results:**

Both resilience and PA significantly predicted QoL. A small but significant interaction effect indicated that PA moderated the resilience–QoL relationship, with stronger benefits among HCPs with lower resilience. Specifically, resilience showed a stronger association with QoL among HCPs with lower levels of PA, whereas this association weakened at higher levels of PA.

**Linking Evidence to Action:**

Healthcare leaders and managers could optimize staff quality of life by implementing interventions that promote positive affect, such as peer‐support systems and recognition initiatives, especially for staff with lower baseline resilience. At the policy level, institutions stand to benefit from adopting broader systemic strategies alongside individual‐focused training to sustain a supportive emotional climate during prolonged crises. Furthermore, future research should utilize longitudinal designs to evaluate the long‐term impact of these interventions on workforce retention.

## 1 Introduction

Healthcare professionals (HCPs) are accustomed to working under stressful conditions, as routine clinical practice involves sustained exposure to high workloads, time pressure, complex decision‐making, and repeated encounters with patient suffering, uncertainty, and life‐threatening situations (Johnson et al. 2020). In Israel, baseline occupational stress among HCPs escalated sharply when the healthcare system entered a state of war on October 7, 2023, while still recovering from the long‐term effects of the COVID‐19 pandemic and armed conflict (Zusman and Andrews 2026). This “dual crisis” was predictable, given prior evidence showing that during periods of extreme healthcare demand, mass casualty events, limited resources, and the breakdown of routine social support systems further intensify the burden on HCPs (Cohen and Rodgers 2020; Thu et al. 2022).

Beyond these systemic pressures, the war generated a layered vulnerability, as HCPs were required to navigate conflicting professional obligations and personal responsibilities toward their families (Zusman and Andrews 2026). While continuing to provide care, they carried a persistent emotional burden stemming from concern for the safety of loved ones, as their home environments were simultaneously characterized by ongoing missile threats and pervasive uncertainty. A convergence of personal threat and professional duty can place additional strain on occupational demands and amplify psychological distress among HCPs (Arias‐Ulloa et al. 2023; Zusman and Andrews 2026).

To deepen understanding of HCPs' experiences in Israel during the overlapping crises of the COVID‐19 pandemic and subsequent armed conflict, a preliminary qualitative study examined factors supporting nurses' cognitive and emotional coping through ten in‐depth interviews (Zusman and Andrews 2026). The findings identified resilience and PA as central psychosocial resources, hypothesized to influence HCPs' QoL under conditions of environmental instability.

Building on these findings, the present study extends this work to a national sample of healthcare professionals (HCPs), offering quantitative evidence on both the independent and interactive contributions of resilience and PA to QoL. By shifting from context‐specific qualitative insights to population‐level analysis, this study addresses an important gap in the literature and informs evidence‐based strategies to support HCPs QoL under prolonged high‐stress conditions.

## 2 Background and Significance

QoL is defined as a broad measure of physical, mental, and social well‐being (WHOQOL Group 1998). While extreme stressors like war and pandemics severely damage the QoL of HCPs (Sarid et al. 2025), resilience acts as a vital protective buffer, often defined as the ability to “bounce back” and adapt to adversity (Tugade and Fredrickson 2004). In military medical personnel, higher resilience was found to be linked to greater job satisfaction and lower burnout and secondary traumatic stress, key determinants of professional QoL (Leners et al. 2014).

One mechanism underlying this protective effect is positive affect (PA), a central component of the resilience, PA, and QoL model. PA serves as an emotional resource that enhances flexible coping and psychological adjustment under stress, buffering the impact of adversity and compensating for lower resilience (Egan et al. 2024; Ong et al. 2006; Fredrickson 2001; Seligman 2011). Resilience supports QoL partly through a greater tendency to experience frequent and intense positive emotions, such as enthusiasm, curiosity, and joy (Watson et al. 1988). Research highlights this utility in high‐pressure settings: for example, physicians who used positive reframing during COVID‐19 showed higher resilience against traumatic stress (Lai et al. 2025), which may indicate a protective effect on their QoL.

Despite the established role of PA and resilience in shielding HCPs, significant gaps remain in understanding how these mechanisms interact to shape the QoL of HCPs during wartime. While existing research often examines the dual relationships between PA and resilience, or resilience and QoL, there is no study to date that explains the interaction between them. Addressing this gap is essential for designing evidence‐based strategies to safeguard HCPs in high‐conflict environments where vulnerability is most acute.

## 3 Purpose and Aims

**FIGURE 1:**
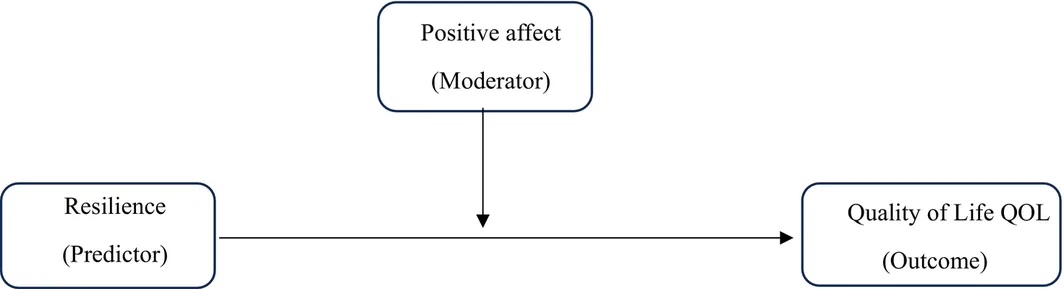
Positive affect was examined as a moderator of the relationship between resilience and quality of life.

Given that the preservation of QoL under the extreme stress of conflict zones has received limited empirical attention, this study aims to investigate the moderating role of PA in the relationship between resilience and QoL. We hypothesize that PA enhances QoL independently of resilience and that elevated levels of PA strengthen the beneficial impact of resilience on overall QoL (Figure 1).

## 4 Theoretical Framework

This study was framed by Fredrickson's (2004) broaden‐and‐build theory, which suggests that positive emotional experiences expand individuals' thought–action repertoires (“broaden”) and, over time, contribute to the development of enduring personal resources (“build”) that support adaptive coping. Within this framework, PA is conceptualized as a dynamic emotional resource that enhances cognitive flexibility and coping under continuous stress, while resilience is viewed as a more stable, accumulated capacity reflecting prior adaptive processes.

This theoretical perspective forms the basis for our moderation hypothesis. Resilience represents the “built” reservoir of stable psychological resources, whereas PA serves as the “broadening” mechanism that activates these resources. We propose that PA moderates the relationship between resilience and QoL by enhancing cognitive flexibility, enabling HCPs to more effectively leverage their existing resilience. In this sense, PA functions as a catalyst: when PA is high, the protective impact of resilience on QoL is amplified, whereas low PA may interrupt the broaden‐and‐build cycle, leaving even resilient individuals vulnerable to the depleting effects of prolonged crisis.

## 5 Methods

### 5.1 Design and Sample

Data were collected from 260 healthcare workers in Israel between May and September 2024. Using a national online cross‐sectional descriptive‐correlational design, this study examined the predictive role of resilience in QoL and the moderating effect of PA among HCPs during a period of intense crisis. Inclusion criteria included healthcare professionals aged 18 years or older who were able to read and write in Hebrew. Participants were recruited by distributing the questionnaire link through social media platforms (e.g., Facebook, WhatsApp) and via hospital email lists targeting nurses, physicians, social workers, and other healthcare professionals. Data were collected using a combination of Qualtrics and Google Forms, as per the guidelines of each ethics committee.

### 5.2 Measures

Data were collected using a demographic questionnaire designed to obtain descriptive information about participants, including gender, age, marital status, education level, religious affiliation, level of religiosity, place of employment, profession, managerial role, department, and years of experience. In addition, resilience was assessed using the Brief Resilience Scale (BRS), QoL was measured with the Multicultural Quality of Life Index (MQLI), and positive affect was evaluated using the Positive and Negative Affect Schedule (PANAS).

The Brief Resilience Scale (BRS; Smith et al. 2008) assesses an individual's perceived ability to recover or “bounce back” from stress. The scale conceptualizes resilience as a unidimensional construct and consists of six items, including both positive and negatively worded statements. Responses are rated on a 5‐point Likert scale ranging from 1 (strongly disagree) to 5 (strongly agree). Average scores range from 1 (low resilience) to 5 (high resilience), interpreted as follows: 1.00–2.99 = low resilience, 3.00–4.30 = normal resilience, and 4.31–5.00 = high resilience. The scale has demonstrated good internal consistency (Cronbach's *α* = 0.80–0.91) and test–retest reliability of 0.69 (Smith et al. 2008) and is widely used in research and clinical settings to examine resilience in relation to stress, coping, and health outcomes. In other studies, Sánchez et al. (2021) reported a Cronbach's *α* of 0.871, and in the present study internal consistency was good (Cronbach's *α* = 0.78).

The multicultural quality of life index (MQLI; Mezzich et al. 2011), is a validated instrument designed to measure perceived quality of life across multiple domains. The MQLI evaluates satisfaction with physical well‐being, psychological/emotional well‐being, self‐care, occupational functioning, interpersonal relationships, social–emotional support, community and services, and overall life satisfaction. It consists of 10 items rated on a Likert‐type scale, with higher scores indicating higher perceived quality of life. The MQLI has demonstrated strong psychometric properties, including reliability, validity, and cross‐cultural applicability, making it suitable for diverse populations in both clinical and research settings. Mezzich et al. (2011) reported a Cronbach's *α* of 0.87, and in the present study, internal consistency was excellent (Cronbach's *α* = 0.92).

The positive and Negative Affect Schedule (PANAS) was developed by Watson et al. (1988). This instrument comprises two 10‐item mood scales designed to measure both positive affect (PA) and negative affect (NA). Positive affect reflects feelings of energy, enthusiasm, and alertness, whereas low PA is characterized by sadness and low energy. In contrast, negative affect reflects distress and unpleasurable feelings. Each item is rated on a Likert‐type scale, and higher scores indicate greater levels of the corresponding affect. The PANAS has demonstrated good psychometric properties, with Watson et al. (1988) reporting strong internal consistency for both subscales. In the present study, internal consistency for positive affect (PA) was excellent (Cronbach's *α* = 0.86). NA was not analyzed for this study because only data from the PA subscale were used.

### 5.3 Statistical Analysis

Statistical analyses were performed using SPSS (version 27) and R (version 4.3.1). Descriptive statistics summarized participant characteristics. Group differences were examined using Wilcoxon rank‐sum and Kruskal–Wallis tests. Associations between variables were analyzed using multivariate linear regression, and interaction effects were tested with the PROCESS macro for SPSS. There were no missing data, and potential confounders were excluded due to negligible effects in univariate analyses with QoL.

### 5.4 Ethical Considerations

This study was approved by the [blind] University Ethics Committee and the [blind] Hospital Ethics Committee and was conducted in accordance with the principles outlined in the Declaration of Helsinki (2013). Participation was voluntary and the survey was completed anonymously; survey‐engine settings prevented any form of tracking. The consent form clearly stated participants' right to withdraw at any time by choosing not to submit their responses and provided the researchers' contact information for questions or for referral to counseling services if needed. Completion and electronic submission of the survey were accepted as evidence of informed consent.

## 6 Results

The initial sample of the study included 293 participants; however, 33 were excluded due to incomplete questionnaires. The final sample, consisting of 260 HCPs, included: primarily nurses, physicians, and others. Most participants were female (79%) and married (79%). The mean age was 49.7 years (SD = 11.8), and the average professional seniority was 22.8 years (SD = 12.4). Educational attainment was high, with many holding BA, MA, or PhD degrees. The majority identified as Jewish (75%), and the mean level of religiosity was moderate (M = 4.03). Over half of participants (52%) reported holding a managerial role.

Differences in QoL, resilience, and PA across sociodemographic groups, including group means, standard deviations, and *p*‐values, are presented in Table 1. In brief, men reported higher QoL and resilience than women, although no gender differences were observed in PA. Higher education levels were associated with greater QoL, resilience, and PA, with participants holding master's and PhD degrees scoring higher than those with bachelor's degrees. In addition, participants in managerial roles reported significantly higher QoL, resilience, and positive affect compared with those without managerial responsibilities. No significant differences were found based on marital status or profession.

**TABLE 1:** Differences in quality of life (QoL), resilience, and positive affect (PA) across sociodemographic groups.

Variable	QoL		Resilience		PA	
	Mean (SD)	*p*	Mean (SD)	*p*	Mean (SD)	*p*
Gender						
Male	7.47 (1.72)	0.02*	3.73 (0.76)	0.04*	3.38 (0.72)	0.91^2^
Female	6.97 (1.65)		3.57 (0.68)		3.38 (0.61)	
Marital status						
Married	7.18 (1.63)	0.15^2^	3.63 (0.68)	0.50^2^	3.42 (0.64)	0.19^2^
Not married	6.69 (1.84)		3.48 (0.79)		3.27 (0.64)	
Profession						
Nurse	6.92 (1.68)	0.66^1^	3.58 (0.70)	0.19^1^	3.41 (0.65)	0.53^1^
Physician	7.3 (1.55)		3.75 (0.75)		3.33 (0.61)	
Else	(7.52)		3.48 (0.56)		3.35 (0.59)	
Education						
Bachelor's	6.62 (1.75)	0.01*^1^	3.37 (0.70)	0.00**^1^	3.26 (0.63)	0.01*^1^
Master's	7.27 (1.55)		3.74 (0.62)		3.50 (0.61)	
PhD	7.46 (1.48)		3.76 (0.69)		3.47 (0.63)	
Managerial role						
No	6.76 (1.76)	0.00**^2^	3.48 (0.72)	0.00**^2^	3.28 (0.62)	0.00**^2^
Yes	7.34 (1.61)		3.77 (0.60)		3.50 (0.63)	

* Correlation is significant at the 0.05 level.

** Correlation is significant at the 0.01 level.

**FIGURE 2:**
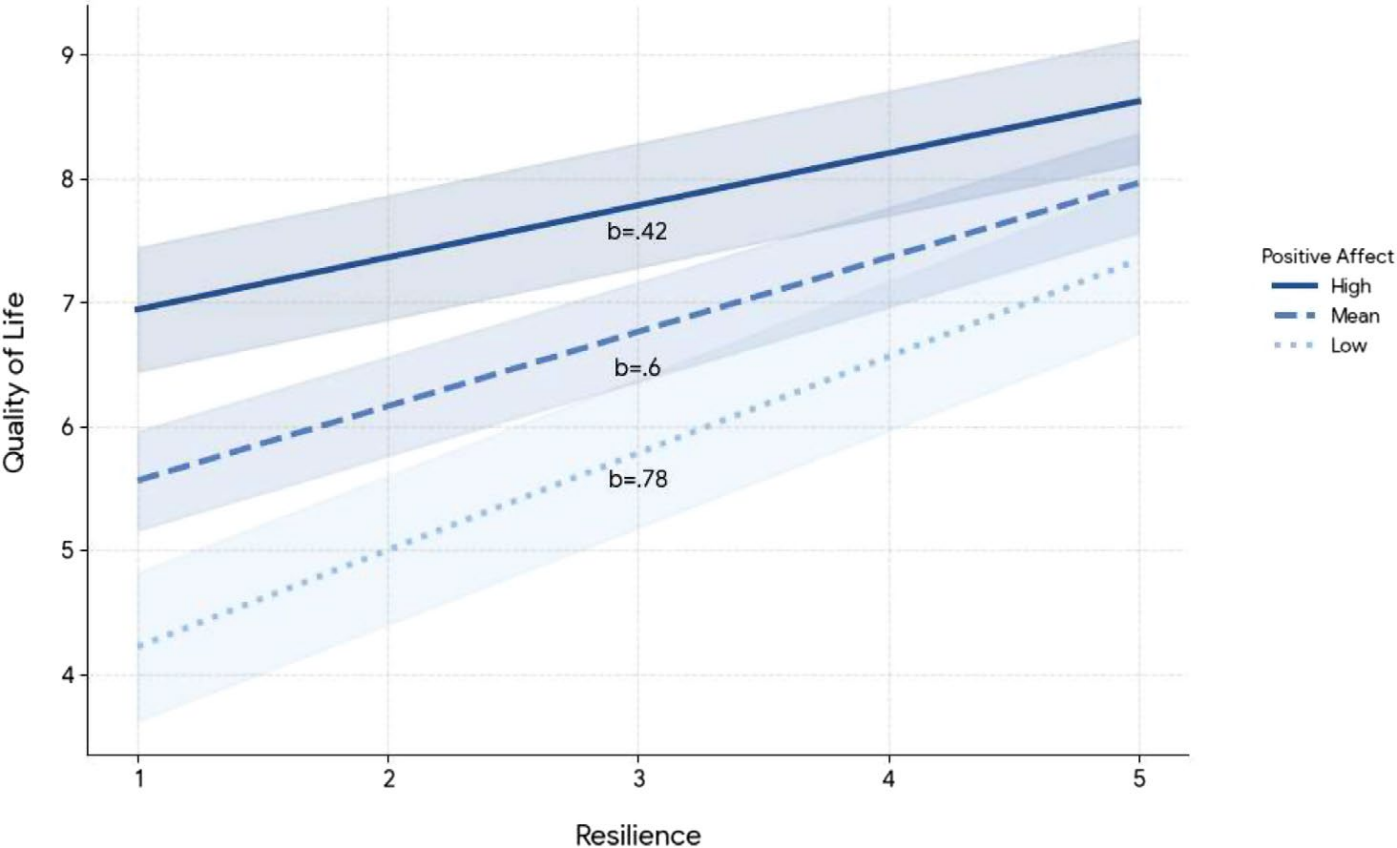
The simple slope plot illustrates a moderation effect, where the relationship between Resilience and QoL changes depending on the level of PA When resilience is low, there is a large gap between the “High” and “Low” positive affect lines. This means that if someone has low resilience, having high Positive Affect makes a meaningful difference in their quality of life (jumping from about a 4.2 to a 7.0). When resilience is high: The gap between “High” and “Low” positive affect is much smaller. This suggests that if someone is already highly resilient, their level of Positive Affect doesn't matter nearly as much; they have a high quality of life either way.

Regarding the strength and direction of the relationships among the study variables, significant positive correlations were found between QoL and resilience, as well as between resilience and PA. The strongest correlation, however, was observed between QoL and PA (see Table 2). Subsequently, a moderation analysis was conducted using a multiple linear regression framework. Both resilience and PA emerged as significant predictors of QoL. Higher levels of resilience (*β* = 0.25, *p* < 0.001) and positive affect (β = 0.53, *p* < 0.001) were associated with better QoL. In addition, a small but statistically significant interaction effect was found (*β* = −0.08, *p* = 0.047), indicating that PA moderated the relationship between resilience and QoL. Specifically, resilience showed a stronger association with QoL among individuals with lower levels of PA, whereas this association weakened at higher levels of PA. Overall, the model explained 48% of the variance in QoL (see Figure 2 for an illustration of the interaction).

**TABLE 2:** Correlations between quality of life, resilience, and positive.

Variable	Quality of life	Resilience	Positive affect
Quality of life	—		
Resilience	0.505**	—	
Positive effect	0.641**	0.438**	—

** *p* < 0.01.

## 7 Discussion

This study examined the moderating role of PA in the relationship between resilience and QoL among HCPs exposed to prolonged and extreme stress during the armed conflict. The findings indicate that both resilience and PA are central to sustaining QoL in high‐strain contexts. Specifically, PA acted as a significant moderator, exerting its most protective influence when resilience was low. This indicates that for less resilient individuals, high PA serves as a critical compensatory resource that prevents a decline in QoL. Conversely, as resilience levels increase, the influence of PA diminishes, suggesting that high resilience provides a stable foundation for well‐being that is less dependent on immediate emotional states.

This interaction underscores that HCPs with limited internal coping resources derive the greatest benefit from elevated PA. Such a pattern is consistent with the broaden‐and‐build theory, which posits that positive emotions broaden cognitive flexibility and facilitate the acquisition of enduring psychological resources (Fredrickson 2004; Tugade and Fredrickson 2004). Evidence from the COVID‐19 pandemic indicates that engagement in pleasurable activities significantly reduces declines in both physical and psychological health (Mulhem et al. 2025). Supporting this perspective, proactive self‐care and adaptive coping strategies have been empirically linked to superior well‐being outcomes among HCPs (Wang et al. 2022). Collectively, these findings suggest that the intentional pursuit of restorative behaviors functions as a primary mechanism through which PA fortifies resilience, thereby safeguarding long‐term QoL during sustained crises Table 3.

**TABLE 3:** Linking Evidence to Action.

Results of this study demonstrate that positive affect plays a meaningful moderating role in the resilience‐quality of life relationship, improving the predictive power of quality‐of‐life models.
Findings indicate that positive affect and resilience contribute to healthcare professionals' quality of life through distinct but complementary pathways during prolonged crisis conditions.
Positive affect may be particularly important for healthcare professionals with lower resilience, suggesting value in strategies that enhance momentary emotional and cognitive resources under acute strain.
Resilience functions as a more durable protective resource, supporting quality of life independent of short‐term emotional fluctuations.
Quality of life interventions might be enhanced by a more individualized approach, adjusting positive affect and resilience supports to match healthcare professionals' coping strengths.

To facilitate these restorative processes at an organizational level, evidence‐based interventions including positive psychology programs, mindfulness training, and cognitive behavioral therapy (CBT) have consistently proven effective in enhancing PA and resilience while alleviating occupational stress (Janitra et al. 2025; Moskowitz et al. 2024). Integrating these interventions into routine training and organizational support systems offers a scalable strategy to strengthen healthcare professionals' psychological resources and protect their QoL. Formalizing these supports can help build a more resilient and adaptable workforce in demanding clinical settings.

In line with this, resilience functions as a robust internal psychological resource that supports effective emotion regulation, buffers the harmful effects of chronic stress, and preserves overall well‐being capacities traditionally enhanced by PA (Tugade and Fredrickson 2004; Yildirim and Arslan 2023). Extending prior research, our findings demonstrate that higher resilience reduces reliance on PA to sustain QoL, underscoring resilience as a self‐sustaining protective mechanism that preserves well‐being under extreme and prolonged stress and highlighting the critical importance of cultivating and maintaining this capacity.

However, resilience is not a fixed trait but a dynamic capacity that can fluctuate or erode over time, particularly under sustained high‐acuity stress (Du et al. 2025). This makes preserving resilience a critical priority in healthcare settings (Hamama et al. 2025; Zalcman et al. 2023). Coaching interventions that focus on skill acquisition and increased self‐awareness have been shown to strengthen resilience among HCPs (Janes et al. 2022; Rosen et al. 2022). By reinforcing these internal capacities, such interventions can help HCPs maintain psychological stability even when positive emotional resources are limited. However, despite their promise, further empirical research is needed to determine their effectiveness in extreme and prolonged crisis contexts, including active conflict environments.

## 8 Limitations and Directions for Future Research

Regardless of the high significance of these findings, several limitations warrant consideration. First, the cross‐sectional design limits our ability to establish causality. While a strong relationship exists between resilience, PA, and QoL, the directional nature of these links remains difficult to determine without longitudinal data. A cross‐sectional approach cannot fully capture the dynamic fluctuations of these variables, specifically the “depletion” of resilience as it is consumed over an extended period of time (Du et al. 2025). Future research should track HCPs over time to examine how their psychological resources drain and recover during prolonged high‐stress conditions.

Second, the sample was drawn from a single national context, limiting generalizability to other healthcare systems or crisis settings. Longitudinal studies are needed to clarify temporal relationships and test whether interventions targeting PA and resilience lead to sustained improvements in resilience and QoL in different environments. Third, because this study was conducted among HCPs experiencing the unique and compounded challenges of both a pandemic and active war, the findings may not be fully generalizable to more stable clinical settings.

Fourth, HCPs were exposed to two major consecutive stressors, the COVID‐19 pandemic and an active war. The cumulative impact of these events may differ from the effects of war exposure alone, limiting the generalizability of the findings to populations experiencing only a single large‐scale stressor.

However, the extreme nature of these stressors provided a unique “stress test” for the resilience‐PA interaction, offering insights that may be applicable to other high‐stakes emergency sectors.

Finally, while this study highlighted the efficacy of PA as a moderator, it did not account for organizational‐level factors such as workload, institutional support, or staffing ratios. Future investigations should adopt a multilevel approach to examine how systemic support structures interact with individual psychological resources to protect healthcare staff's well‐being.

## 9 Conclusion

This study provides critical insights into the psychological mechanisms that safeguard the QoL of HCPs during the armed conflict. By examining the interplay between resilience and PA, we have demonstrated that while both factors are essential for maintaining well‐being, their roles are distinct and context‐dependent. Specifically, our findings suggest that while PA serves as a vital compensatory resource for individuals with lower resilience, its influence diminishes as resilience levels increase. In high‐resilience individuals, resilience functions as a more autonomous protective factor, stabilizing QoL independently of immediate emotional states.

## Funding

This work was funded by the Hadassah Research Center and a grant from the Silverstein Fund for help with translation and transcription services, payment for software support services, and statistical support.

## Ethics Statement

Ethics Committee of Hebrew University Jerusalem (#31102022) and the Hadassah Medical Center Helsinki Committee (#0024–24‐HMO).

## Consent

Informed consent was obtained via tacit approval to submit the online survey. The statistics were checked prior to submission by an expert statistician, and state their name and email address: Halit Kantor, MPH Biostatistician halitkantor18@gmail.com. Data is available for review as requested.

## Conflicts of Interest

The authors declare no conflicts of interest.

## Data Availability

The data that support the findings of this study are available on request from the corresponding author. The data are not publicly available due to privacy or ethical restrictions.
